# microRNAs are differentially expressed in equine plasma of horses with osteoarthritis and osteochondritis dissecans versus control horses

**DOI:** 10.1371/journal.pone.0297303

**Published:** 2024-02-23

**Authors:** Joshua Antunes, Ramés Salcedo-Jiménez, Starlee Lively, Pratibha Potla, Nathalie Coté, Marie-Soleil Dubois, Judith Koenig, Mohit Kapoor, Jonathan LaMarre, Thomas Gadegaard Koch

**Affiliations:** 1 Department of Biomedical Sciences, Ontario Veterinary College, University of Guelph, Guelph, Ontario, Canada; 2 Department of Clinical Studies, Ontario Veterinary College, University of Guelph, Guelph, Ontario, Canada; 3 Osteoarthritis Research Program, Division of Orthopedics, Schroeder Arthritis Institute, University Health Network, Toronto, Ontario, Canada; Fondazione Don Carlo Gnocchi, ITALY

## Abstract

Osteoarthritis (OA) is a leading cause of lameness in horses with no effective disease-modifying treatment and challenging early diagnosis. OA is considered a disease of the joint involving the articular cartilage, subchondral bone, synovial membrane, and ligaments. Osteochondritis dissecans (OCD) is a joint disease consisting of focal defects in the osteochondral unit which may progress to OA later in life. MicroRNAs (miRNAs) have been recognized as small non-coding RNAs that regulate a variety of biological processes and have been detected in biological fluids. MiRNAs are currently investigated for their utility as biomarkers and druggable targets for a variety of diseases. The current study hypothesizes that miRNA profiles can be used to actively monitor joint health and differences in miRNA profiles will be found in healthy vs diseased joints and that differences will be detectable in blood plasma of tested horses. Five horses with OA, OCD, and 4 controls (C) had blood plasma and synovial fluid collected. Total RNA, including miRNA was isolated before generating miRNA libraries from the plasma of the horses. Libraries were sequenced at the Schroeder Arthritis Institute (Toronto). Differential expression analysis was done using DESeq2 and validated using ddPCR. KEGG pathway analysis was done using mirPath v.3 (Diana Tools). 57 differentially expressed miRNAs were identified in OA vs C plasma, 45 differentially expressed miRNAs in OC vs C plasma, and 21 differentially expressed miRNAs in OA vs OCD plasma. Notably, miR-140-5p expression was observed to be elevated in OA synovial fluid suggesting that miR-140-5p may serve as a protective marker early on to attenuate OA progression. KEGG pathway analysis of differentially expressed plasma miRNAs showed relationships with glycan degradation, glycosaminoglycan degradation, and hippo signaling pathway. Interestingly, ddPCR was unable to validate the NGS data suggesting that isomiRs may play an integral role in miRNA expression when assessed using NGS technologies.

## Introduction

Joint disease and osteoarthritis (OA) are leading causes of lameness in horses [1]. To date, there is no effective disease modifying treatment of OA [2]. OA is a disease of the joint organ involving articular cartilage, subchondral bone, synovial membrane, and ligaments [3]. Osteochondrosis dissecans is another joint condition that commonly affects horses and is characterized by lesions resulting from abnormal endochondral ossification, its exact pathogenesis remains largely unknown [4]. Osteochondritis dissecans (OCD) is a potential manifestation of this condition involving the detachment of osteochondral fragments into the joint space which can cause inflammation, pain, and lameness. In both OA and OCD, there is a potentially large therapeutic window, but the trouble lies in being able to detect the diseases at early stages. Biomarkers for OA and OCD are needed for early disease detection so that more timely intervention can occur as well as for measuring the response to emerging therapies.

MicroRNAs (miRNA; miR) are small, non-coding RNAs that regulate a variety of biological processes in a post-transcriptional manner [5]. They also have been identified as attractive candidate biomarkers in a variety of diseases. MiRNAs are uniquely stable within collected biofluid samples when compared to other RNA molecules, even at room temperature for up to 48 hours [6]. Typically, proteins have been utilized as biomarkers, but miRNAs have key attributes that also make them appealing candidates. First, miRNAs are less species-specific compared to proteins due to greater phylogenetic conservation of miRNA sequences, which often allows the same miRNAs of interest to be applied as potential biomarkers across species [7,8]. Second, tissue-specific miRNAs exist that can be detected in readily obtained blood and other biofluids. Thus, miRNAs are ideal biomarker candidates, particularly of tissue-specific diseases.

MicroRNAs have been associated with various human joint diseases, but the miRNA profiles appear to differ in a disease-specific manner. Plasma and synovial fluid samples from patients suffering from rheumatoid arthritis or OA exhibited different miRNA profiles from each other as well as compared to controls [9]. Altered miRNA profiles have been associated with OA. For instance, low expression of miR-146a in cartilage has been associated with OA [10] and miR-140 has been observed to attenuate joint degradation in a surgically induced rat OA model via intra-articular (IA) injection [11]. miR-181a and miR-4454 was found to be elevated in human facet joint (FJ) OA cartilage tissue and miR-181a inhibitors were shown to suppress the expression of catabolic, inflammation, and cell death markers [12]. A follow-up study has shown that IA injection of a miR-181a-5p antisense oligonucleotide (ASO) in an induced rat FJ OA and a mouse knee OA model attenuated cartilage destruction [13].

The understanding of the role miRNAs play in equine joint health is currently limited. Based on the current literature surrounding miRNA involvement in the human joint disease and rodent models, miRNAs contained within plasma and synovial fluid hold promise as future biomarkers to identify OA and OCD. We hypothesize unique miRNA signatures can distinguish between horses with control vs diseased joints in both plasma and synovial fluid. To test this hypothesis, next generation sequencing (NGS) was utilized to examine the blood plasma of horses with OA, OCD, and control horses (C). To date, few sequencing studies have been completed in an equine model encompassing synovial fluid and blood plasma [14,15]. This study is among the first to compare miRNAs in horses with diseased joints vs controls utilizing NGS.

## Materials and methods

### Animals

Five horses with OA, 5 horses with OCD, and 4 control horses were enrolled in the sequencing portion of this study. Categorical data is summarized in Table 1. OA and OCD status was confirmed using radiographic and arthroscopic assessment from an equine surgeon at Ontario Veterinary College Large Animal Surgery. Diseased joints in OA horses were sampled from radiocarpal (3), metacarpophalangeal (1) and metatarsophalangeal (1) joints (number of joints sampled in brackets). In OCD horses, the tibiotarsal (4) and metatarsophalangeal (1) joints were sampled (Table 1). Horses from the Arkell research heard were considered as controls. Those with joint effusion, bone abnormalities, prior history of lameness, or chronic inflammatory disease, as assessed by an equine veterinarian (Dr. Ramés Salcedo) for signs of lameness during a walk and trot on a straight line, were exclude from the control group. The inclusion criteria for the control horses were the absence of lameness at the trot on a straight line on subjective assessment and negative flexion tests of the upper and lower limbs. Synovial fluid was aseptically collected from the following joints of control horses: tibiotarsal (2), metatarsophalangeal (1), and metacarpophalangeal (1). Routine synovial fluid cytology was performed at the OVC Animal Health Laboratory. All animal work was approved by the Animal Care Committee at the University of Guelph (Animal Care Protocol #4061). Control horses were sedated using xylazine before anesthetized with Butorphanol tartrate. Client horses underwent routine arthroscopy and a fraction of blood and synovial fluid was collected following verbal consent.

**Table 1:** Summary of patients in the study population.

	C			OA			OCD		
Sex	F(3)	MC(0)	M(1)	F(1)	MC(3)	M(1)	F(5)	M(0)	M(0)
Age, Mean (SD)	17 ± 3.3			3.29 ± 0.7			1.68 ± 0.5		
Breed	CC(1), DC(1), TB(1), SB(1)			TB(1), SB(2), QH(1)			SB(5)		
Sampled Joints									
Radiocarpal	N/A			3			NA		
Tibiotarsal	2			N/A			4		
Metacarpophalangeal	1			1			N/A		
Metatarsophalangeal	1			1			1		

C–Control Horses; OA–Osteoarthritis; OCD–Osteochondritis dissecnas; F–Female; MC–Male Castrated; M–Male; CC–Clydesdale Cross; DC–Draft Cross; TB–Thoroughbred; SB–Standardbred; QH–Quarter Horse.

### Sample collection

Synovial fluid and blood were collected from client-owned horses with either OA or OCD undergoing joint arthroscopy. Control horses that met the criteria previously described were identified within horses at The Arkell Equine Research Station under the Ontario Ministry of Agriculture and Rural Affairs (OMAFRA) for which researchers at the University of Guelph (UofG) have access to through The Ontario Agri-Food Innovation Alliance program which is a partnership between OMAFRA and UofG. Synovial fluid was collected via joint aspiration following sedation in control horses. Blood was also collected at this time and both fluids being placed immediately on ice. Synovial fluid was spun down at 3,500 x *g* for 5 minutes to remove any cells and debris. The supernatant was collected and flash frozen in liquid nitrogen and stored at -80°C. Equine venous blood was collected into EDTA coated vacutainers and centrifuged for 15 minutes at 2,000 x *g* to separate blood plasma. The plasma was collected and flash frozen in liquid nitrogen and stored at -80°C.

### miRNA isolation and library preparation

Total RNA, including miRNA was isolated from both blood plasma and synovial fluid using the Serum/Plasma Advanced Kit (Qiagen, Hilden, Germany). To combat viscosity, samples were vortexed for 25 seconds immediately after the precipitation buffer was added. The remainder of the protocol was carried out according to the manufacturer’s instructions. miRNA was eluted in 20 μL of RNAse free water, flash frozen in liquid nitrogen, and stored in -80°C.

Extracted plasma miRNA (5 μL) was used to prepare miRNA sequencing libraries using QIAseq miRNA Library Kit (Qiagen) as per manufacturer instructions. Resulting libraries underwent quality control (QC) using TapeStation 4150 (Agilent) with DNA 1000 assay kit (Agilent). All prepared libraries used for sequencing showed a clear and prominent miRNA library peak at the expected 176–186 bp range. Libraries also underwent Qubit quantification to determine miRNA library concentrations for accurate loading onto the flow cell for sequencing.

### NGS

Following QC, plasma libraries were sequenced on a NextSeq 550 using a NextSeq 500/550 High Output Kit v2.5 at the Schroeder Arthritis Institute (Toronto). Initial processing of the data was done on the University Health Network (UHN) cloud computer. Demultiplexing of basecall files was performed, UMI tags were extracted, and adapters were trimmed (UMI-tools v0.5.5). Reads that were too short (<18 bp) or too long (>30 bp) were discarded. Reads were aligned to horse mature miRNAs (miRBase v22.1) and horse reference genome (EquCab3.0) using a custom pipeline previously described in detail [16]. All aligned reads were deduplicated based on extracted UMI tags. Counting of these UMI read tags was used to generate the matrix of raw miRNA expression levels.

To analyze sequencing data, a Linux system running Ubuntu 16.04 and 32GB of RAM was used for all statistical analysis. A Principal Component Analysis was performed on each comparison group to ensure comparison groups are uncorrelated with each other. DE analysis was performed on R 3.6.1 in RStudio 1.2.5 using the DESeq2 Bioconductor package [17]. Count data was transformed to the log2 scale prior to heat map generation. Pheatmap was used to generate all heat maps shown only showing the miRNAs differentially expressed with an adjusted p-value < 0.05. EnhancedVolcano was used to generate heat maps with an LFC cut-off of 1.5 and a p-value cut-off of 0.05 where adjusted p-value was used in its place.

### ddPCR validation

miRNAs were chosen based on the level of differential expression (DE) observed in the sequencing data (log2FC > 1.3 and *P* < 0.05) and a brief literature review of the DE miRNAs focusing on their potential role in joint disease. The selected miRNAs for validation were miR-140-5p, miR-181a, miR-196b, miR-20a, and miR-486-3p. miRNAs were isolated as previously described above. 3 μL of RNA was reverse transcribed in 20 μL reactions using the miRCURY LNA RT Kit (Qiagen) as per manufacturer instructions. The resulting cDNA was diluted 1:30 in preparation for ddPCR analysis. ddPCR assays were prepared in 20 μL reactions containing 10 μL QX200 ddPCR EvaGreen Supermix (Bio-Rad, Hercules, California, USA), 2 μL miRCURY LNA Primer (Qiagen, Toronto, ON, Canada) and 8 μL diluted cDNA. Samples were loaded on a cartridge for droplet generation using 70 μL QX200 Droplet Generation Oil for EvaGreen (Bio-Rad). Cartridges were then placed in a QX200 droplet generator (Bio-Rad). Following droplet generation, the droplets were transferred to a 96-well PCR plate using a multichannel pipette and the plate was heat sealed before being placed in a thermal cycler as follows: 95°C for 5 minutes, 40 cycles of 95°C for 30 seconds and 52.7°C for 1 minute, 4°C for 5 minutes, 90°C for 5 minutes, and an infinite hold of 4°C. Plates were held overnight and read the following morning on a QX200 Droplet Reader (Bio-Rad). Analysis was performed using QuantaSoft Analysis Pro software (Bio-Rad).

### Statistics

ddPCR data was analyzed using total copy number in SAS 9.4 after consultation with a statistician. Data was log transformed and checked for normality and multiple variances. Any identified outliers were removed following a Grubs test. Each miRNA data set then underwent ANOVA and multiple comparisons tests before reporting p-values.

## Results

### miRNA NGS of equine plasma

**Fig 1:**
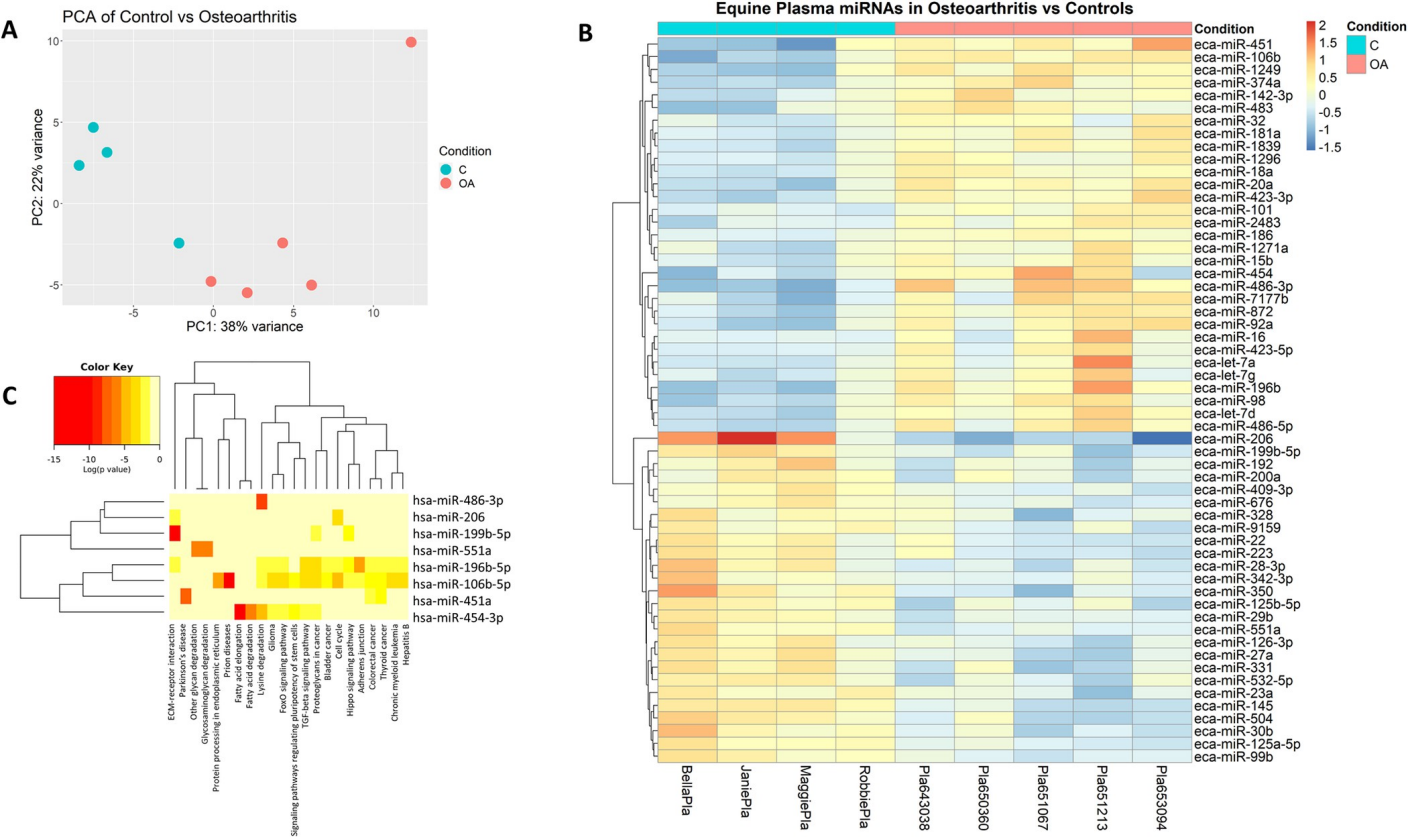
NGS analysis of OA vs C equine plasma. a) PCA plot of C and OA equine blood plasma groups; b) Heatmap of differentially expressed miRNAs in OA vs C plasma. Towards red indicates a fold increase; towards blue represents a fold decrease; c) Pathway analysis using miRPath v.3 and Tarbase v7.0 assessing derived interactions and indicated pathways to have predicted interactions with miRNAs identified to be differentially expressed.

**Fig 2:**
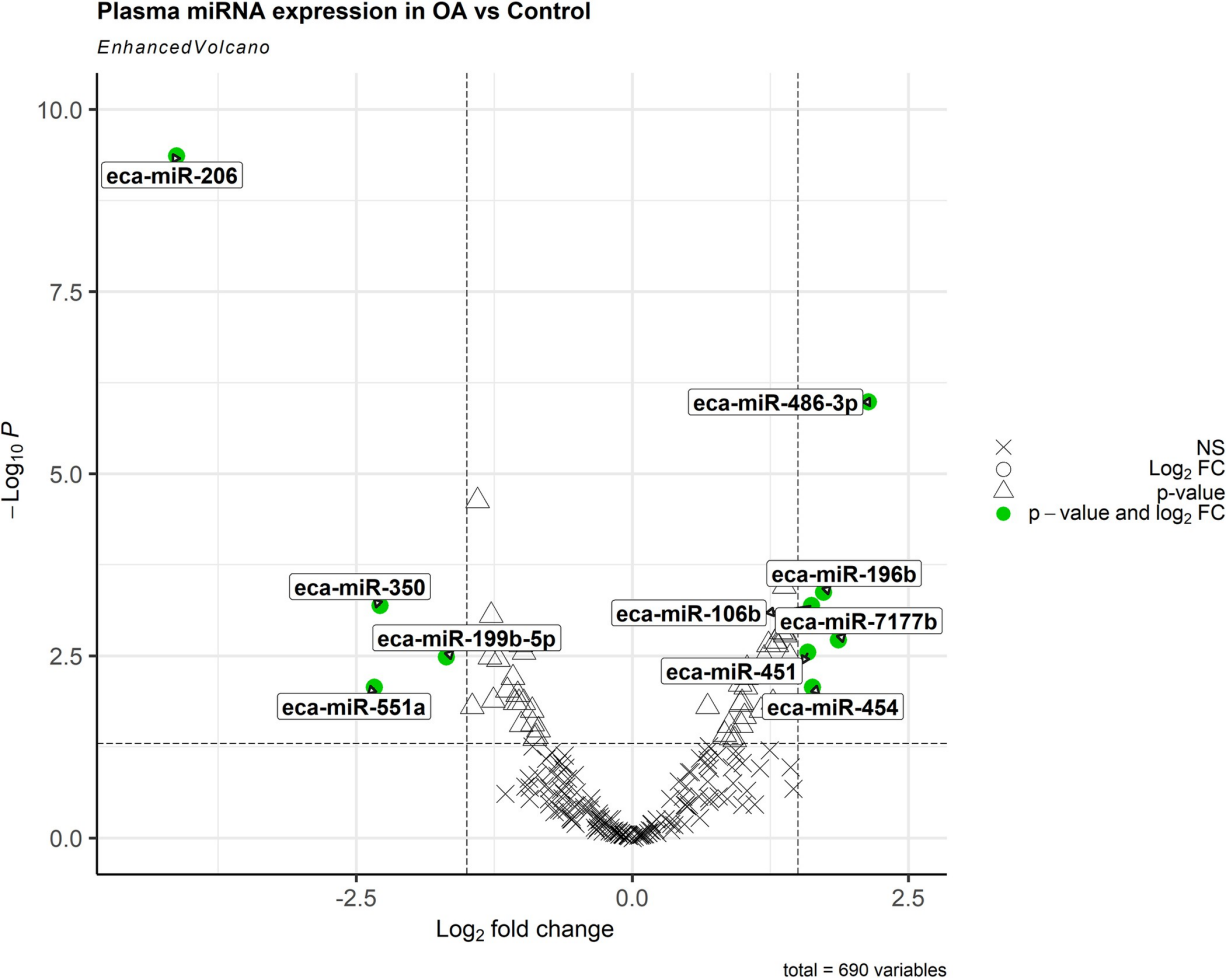
Volcano Plot of OA vs C plasma; LFC cut off = 1.5 and p-value cut off = 0.05; adjusted p-value was used; miRNAs marked in red are those shown to meet both the LFC and p-value cut offs.

In total, 46,766,004 reads were aligned to known mature equine miRNAs. miRNA profiles found in blood plasma of C, OA, and OCD horses were assessed using NGS. Principle component analysis (PCA) demonstrated separation between groups and 57 DE miRNAs were identified in OA vs C (p < 0.05) (Fig 1A and 1B). Of these, 31 miRNAs were upregulated and 26 were downregulated. miR-199b-5p, miR-206 miR-350, and miR-551a were downregulated by > 1.5-log2FC, whereas miR-106b, miR-196b, miR-451, miR-454, miR-486-3p, and miR-7177b were upregulated by > 1.5-log2FC (Figs 1B and 2; Table 2).

**Table 2:** Statistical data of differentially expressed miRNAs equine plasma of OA vs C with a 1.5-log2FC or greater change in expression (p < 0.05).

	baseMean	log2FoldChange	lfcSE	stat	pvalue	padj
*Upregulated*						
eca-miR-486-3p	3663.3	2.141	0.3744	5.720	1.068E-08	1.036E-06
eca-miR-196b	790.52	1.735	0.3946	4.396	1.101E-05	0.0004274
eca-miR-106b	94.371	1.626	0.3843	4.230	2.336E-05	0.0006473
eca-miR-7177b	38.079	1.869	0.4856	3.849	0.0001186	0.001918
eca-miR-451	6226.0	1.590	0.4389	3.622	0.0002920	0.002833
eca-miR-454	56.625	1.632	0.5126	3.184	0.001453	0.008545
*Downregulated*						
eca-miR-206	55.117	-4.127	0.5880	-7.019	2.240E-12	4.346E-10
eca-miR-350	24.407	-2.282	0.5359	-4.259	2.057E-05	0.0006473
eca-miR-199b-5p	29.257	-1.683	0.4712	-3.571	0.0003560	0.003289
eca-miR-551a	7.0618	-2.335	0.7317	-3.191	0.001415	0.008545

baseMean: Mean of normalized counts; log2FoldChange: Log2 fold change; lfcSE: Standard error.

stat: Wald statistic; pvalue: Wald test p-value; padj: BH adjusted p-value.

**Fig 3:**
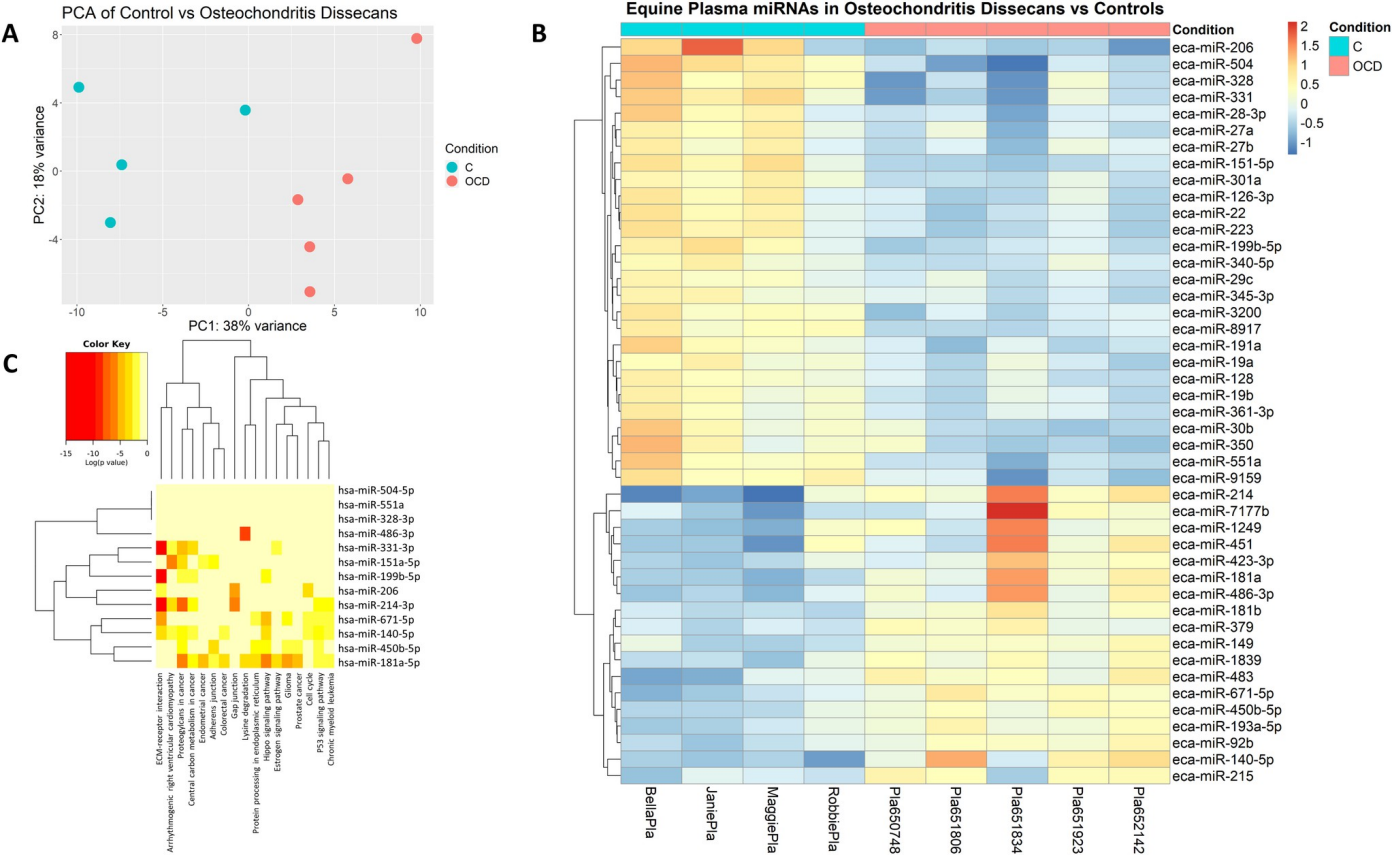
NGS analysis of OCD vs C equine plasma. a) PCA plot of C and OCD equine blood plasma groups; b) Heatmap of differentially expressed miRNAs in OCD vs C plasma. Towards red indicates a fold increase; towards blue represents a fold decrease; c) Pathway analysis using miRPath v.3 and Tarbase v7.0 assessing derived interactions and indicated pathways to have predicted interactions with miRNAs identified to be differentially expressed.

**Fig 4:**
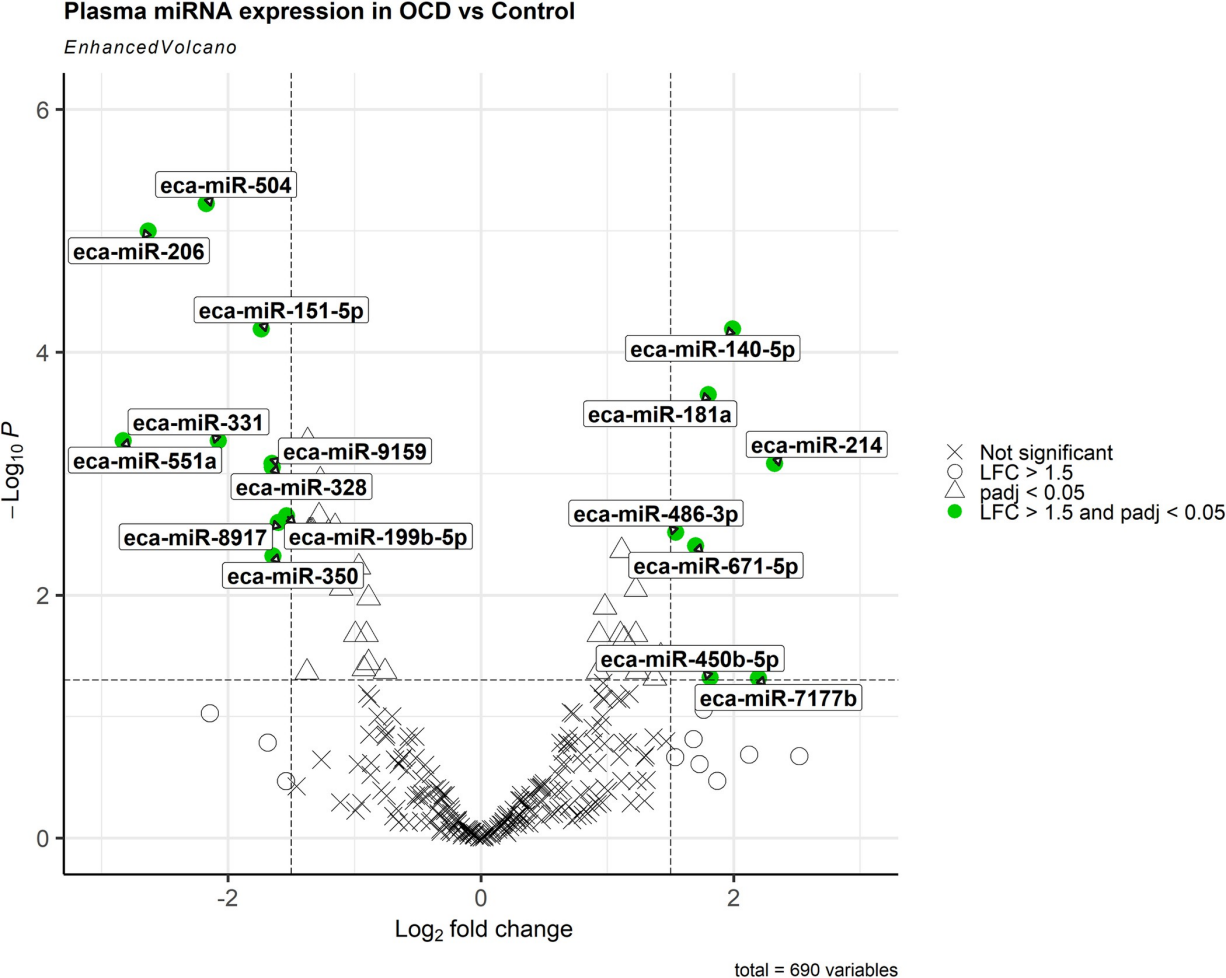
Volcano Plot of OCD vs C plasma; LFC cut off = 1.5 and p-value cut off = 0.05; adjusted p-value was used; miRNAs marked in red are those shown to meet both the LFC and p-value cut offs.

45 DE miRNAs were also identified in OCD horses relative to C and PCA analysis showed separations between the compared groups (Fig 3A and 3B). Of these, 18 miRNAs were upregulated and 27 were downregulated (p<0.05). miR-140-5p, miR-181a, miR-214, miR-486-3p, miR-671-5p, miR-7177b, and miR-450b-5p were all upregulated by > 1.5-log2FC. miR-151-5p, miR-199b-5p, miR-206, miR-328, miR-331, miR-350, miR-504, miR-551a, miR-8917, and miR-9159 were downregulated by > 1.5-log2FC (Figs 3B and 4; Table 3).

**Table 3:** Statistical data of differentially expressed miRNAs equine plasma of OCD vs C with a 1.5-log2FC or greater change in expression (p < 0.05).

	baseMean	log2FoldChange	lfcSE	stat	pvalue	padj
*Upregulated*						
eca-miR-140-5p	334.04	1.991	0.4068	4.896	9.786E-07	6.435E-05
eca-miR-181a	428.65	1.800	0.3914	4.598	4.256E-06	0.0002238
eca-miR-214	81.599	2.324	0.5582	4.163	3.135E-05	0.0008245
eca-miR-486-3p	3789.7	1.543	0.4204	3.670	0.0002427	0.003039
eca-miR-671-5p	22.181	1.701	0.4735	3.591	0.0003289	0.003932
eca-miR-450b-5p	7.9652	1.813	0.6818	2.660	0.007824	0.04785
eca-miR-7177b	65.321	2.196	0.8308	2.644	0.008204	0.04854
*Downregulated*						
eca-miR-504	191.76	-2.172	0.3886	-5.590	2.274E-08	5.981E-06
eca-miR-206	91.730	-2.630	0.4894	-5.375	7.674E-08	1.009E-05
eca-miR-151-5p	80.450	-1.737	0.3539	-4.908	9.217E-07	6.435E-05
eca-miR-331	48.510	-2.075	0.4763	-4.358	1.315E-05	0.0005369
eca-miR-551a	10.065	-2.829	0.6527	-4.334	1.463E-05	0.0005369
eca-miR-9159	58.684	-1.651	0.3946	-4.184	2.867E-05	0.0008245
eca-miR-328	1395.0	-1.649	0.3997	-4.125	3.703E-05	0.0008854
eca-miR-199b-5p	44.539	-1.535	0.3983	-3.853	0.0001165	0.002229
eca-miR-8917	20.073	-1.601	0.4213	-3.801	0.0001444	0.002531
eca-miR-350	41.189	-1.644	0.4673	-3.518	0.0004346	0.004763

baseMean: Mean of normalized counts; log2FoldChange: Log2 fold change; lfcSE: Standard error.

stat: Wald statistic; pvalue: Wald test p-value; padj: BH adjusted p-value.

**Fig 5:**
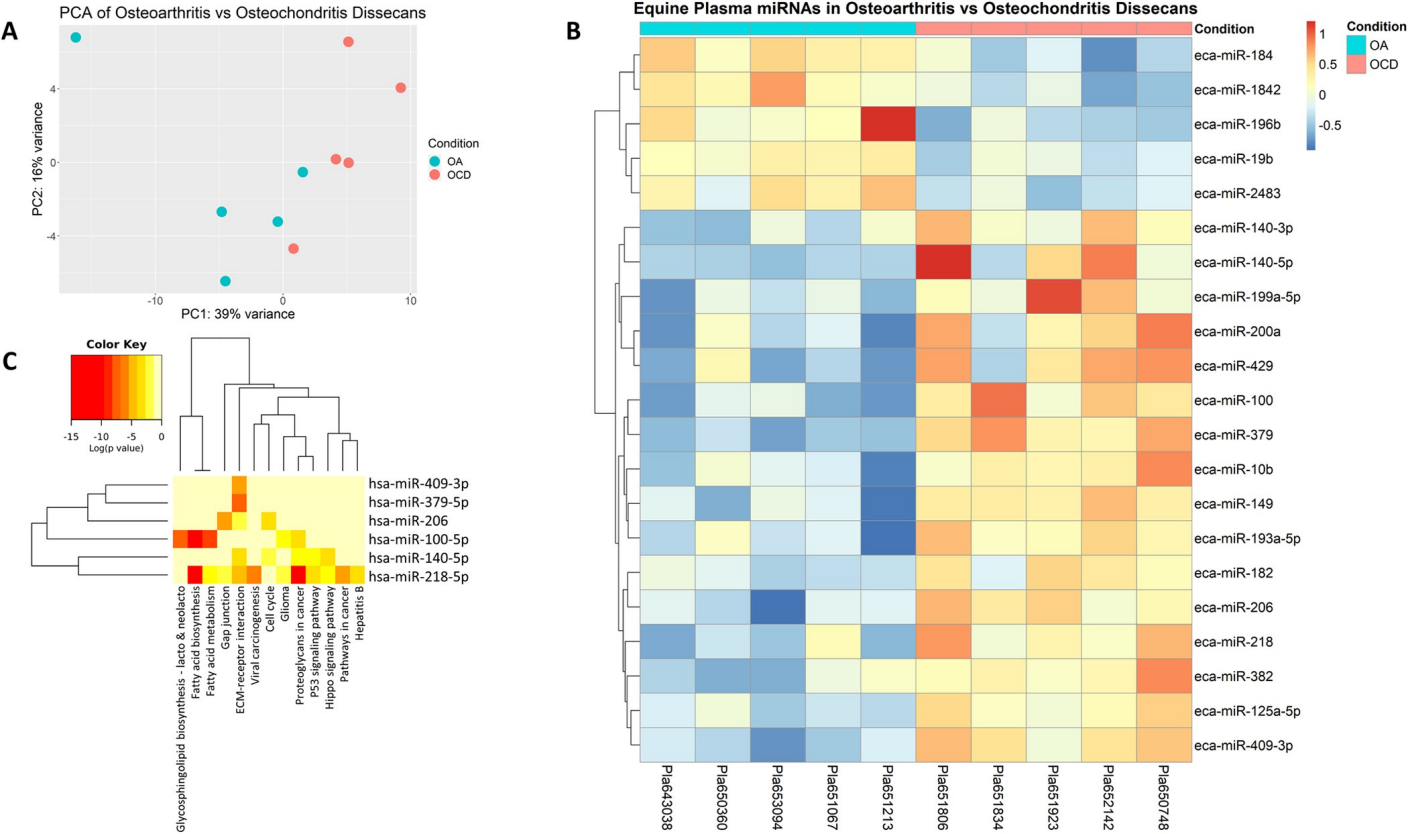
NGS analysis of OA vs OCD equine plasma. a) PCA plot of OA and OCD equine blood plasma groups; b) Heatmap of differentially expressed miRNAs in OA vs OCD plasma. Towards red indicates a fold increase; towards blue represents a fold decrease; c) Pathway analysis using miRPath v.3 and Tarbase v7.0 assessing derived interactions and indicated pathways to have predicted interactions with miRNAs identified to be differentially expressed.

**Fig 6:**
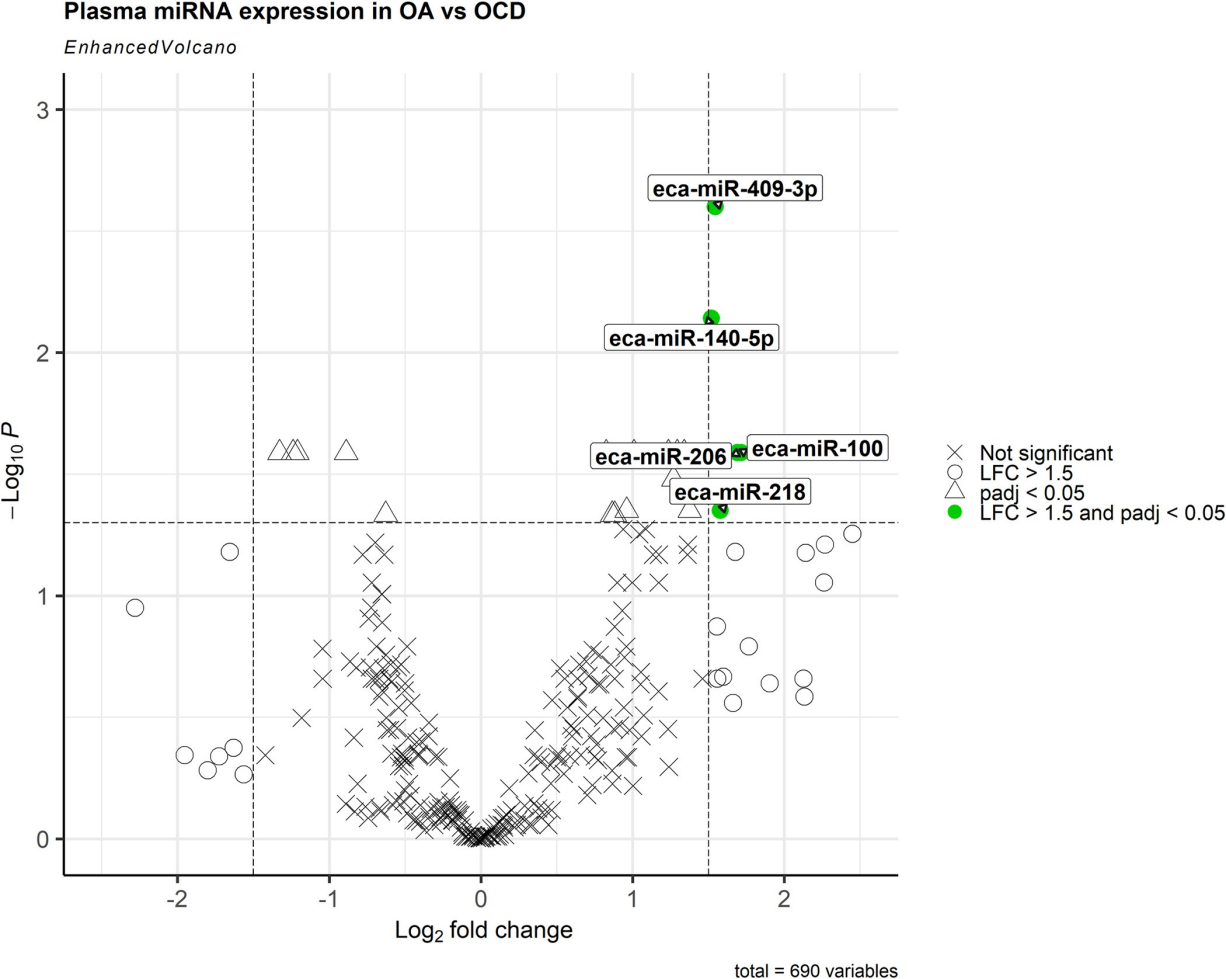
Volcano Plot of OA vs OCD plasma; LFC cut off = 1.5 and p-value cut off = 0.05; adjusted p-value was used; miRNAs marked in red are those shown to meet both the LFC and p-value cut offs.

When the miRNA plasma profiles of OA and OCD horses were compared, PCA anaysis showed some separation between groups and 21 differentially expressed miRNAs were identified (p < 0.05) (Fig 5A and 5B). Of these, 16 were upregulated and 5 were downregulated in OA vs OCD. miR-100, miR-140-5p, miR-206, miR-218, miR-379, and miR-409-3p were significantly upregulated by >1.5-log2FC in OA vs OCD (Figs 5B and 6; Table 4).

**Table 4:** Statistical data of differentially expressed miRNAs equine plasma of OA vs OCD with a 1.5-log2FC or greater change in expression (p < 0.05).

	baseMean	log2FoldChange	lfcSE	stat	pvalue	padj
*Upregulated*						
eca-miR-379	12.092	2.527	0.5700	4.433	9.272E-06	0.002494
eca-miR-409-3p	34.499	1.546	0.3613	4.280	1.869E-05	0.002514
eca-miR-140-5p	272.48	1.521	0.3858	3.943	8.050E-05	0.007218
eca-miR-100	20.888	1.717	0.5299	3.240	0.001196	0.02582
eca-miR-206	14.867	1.692	0.4976	3.399	0.0006752	0.02582
eca-miR-218	14.197	1.578	0.5308	2.972	0.002956	0.04461

baseMean: Mean of normalized counts; log2FoldChange: Log2 fold change; lfcSE: Standard error.

stat: Wald statistic; pvalue: Wald test p-value; padj: BH adjusted p-value.

### ddPCR validation of differentially expressed (DE) miRNAs in plasma and synovial fluid

**Fig 7:**
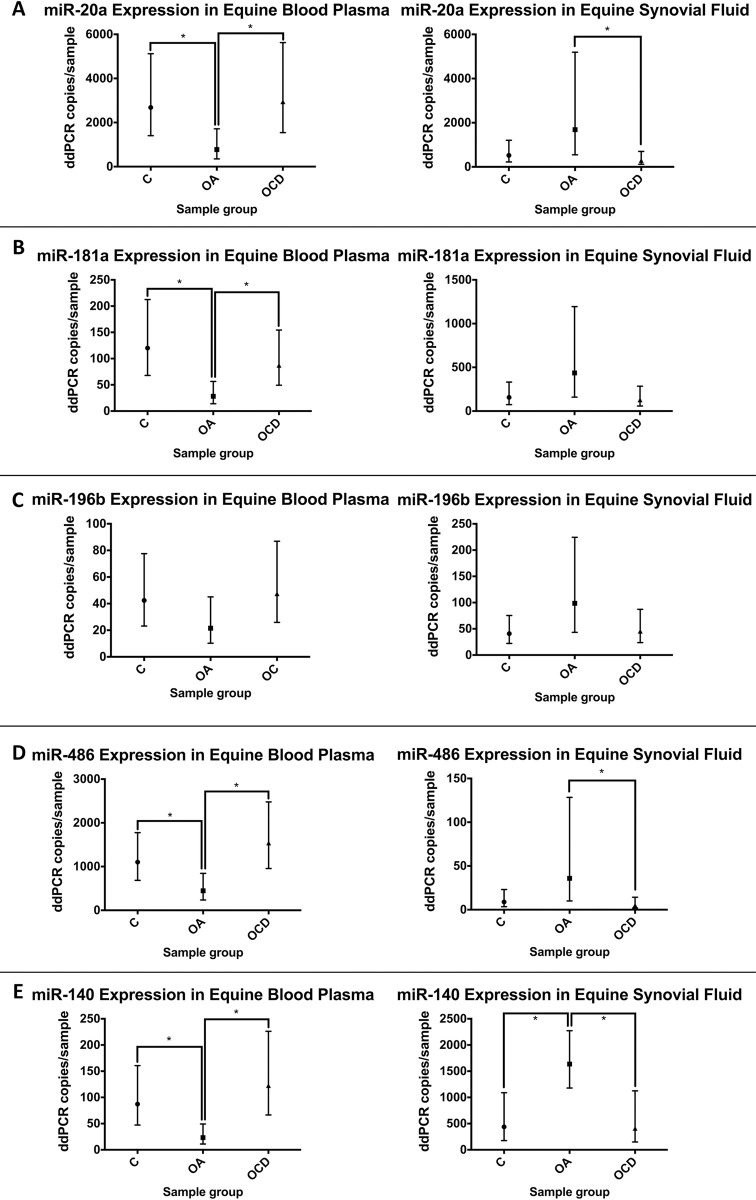
ddPCR analysis of miRNA expression in blood plasma and synovial fluid. a) miR-20a; b) miR-181a; c) miR-196b; d) miR-486; and e) miR-140. (*p<0.05).

Five miRNAs were selected for validation—miR-140-5p, miR-181a, miR-196b, miR-20a, and miR-486-3p –based on a combination of their level of differential expression observed in NGS and a brief literature review. A paired synovial fluid cohort was added to compliment the plasma cohort to compare plasma vs synovial miRNA expression. The sequencing cohort was expanded upon by including additional control and diseased horses (C n = 9; OA n = 6; OCD n = 9). Two identified outliers were removed among the synovial fluid groups resulting in C n = 9, OA n = 5, OCD n = 8. In the plasma samples, one outlier was removed in the OA group for miR-486. miR-140-5p showed a significant difference between groups for both plasma (p>F = 0.0055) and synovial fluid samples (p>F = 0.0051). When examining the differences in miR-140-5p expression between groups, OA vs C plasma showed a 3.740 fold decrease (p = 0.0099), OA vs OCD plasma showed a 5.262 fold decrease (p = 0.0018), OA vs C synovial fluid showed a 3.733 fold increase (p = 0.0103), and OA vs OCD synovial fluid showed a 3.989 fold increase (p = 0.0133) (Fig 7E). miR-181a showed a significant difference in expression in the plasma groups (p>F = 0.0093) but not in the synovial fluid groups (p>F = 0.1429). When comparing the differences in miR-181a expression between groups, OA vs C plasma showed a 4.277 fold decrease in expression (p = 0.0030) and OA vs OCD plasma showed a 3.107 fold decrease in expression (p = 0.0162) (Fig 7B). No significant differences of miR-196b expression were found in plasma (p>F = 0.2188) or synovial fluid (p>F = 0.1991) groups (Fig 7C). miR-20a showed significant differences in expression in plasma (p>F = 0.0273) but not the synovial fluid (p>F = 0.0578) groups. When comparing the differences in miR-20a expression within these groups, OA vs C plasma showed a 3.451 fold decrease in expression (p = 0.0199) and OA vs C plasma showed a 3.790 fold decrease in expression (p = 0.0131). The differences between synovial groups were found to be insignificant. However, despite failing the F test, synovial miR-20a trended towards an increase in expression in OA vs OCD synovial fluid groups (Fig 7A). miR-486-3p was observed to have a significant difference in expression in plasma (p>F = 0.014) but not in synovial fluid (p>F = 0.0665) groups. Comparisons within these groups showed OA vs C plasma to have a 2.4711 fold decrease in expression (p = 0.028) and OA vs OCD plasma to have a 3.451 fold decrease in expression (p = 0.0041). While the synovial groups were not shown to be significant, when examining the differences between synovial fluid groups OA vs OCD trended towards showing an increase in expression (Fig 7D).

Overall, miR-20a demonstrated decreased expression in OA vs OCD and C in equine plasma but increased expression in OA vs OCD synovial fluid (Fig 7A). miR-181a showed decreased expression in OA vs OCD and C plasma and no significant changes in synovial fluid (Fig 7B). miR-196b showed no significant changes of expression in plasma or synovial fluid (Fig 7C). miR-486 demonstrated decreased expression in OA vs OCD and C plasma and increased expression in OA vs OCD synovial fluid (Fig 7D). miR-140 showed a decrease of expression in OA vs OCD and C plasma but increased expression in OA vs OCD and C synovial fluid (Fig 7E).

### Pathway analysis

Following NGS analysis, pathways and potential targets of DE miRNA in plasma were predicted using mirPath v.3 (Diana Tools) with the human miRNA analogues to the equine miRNAs identified. Human and equine miRNA sequences similarities were confirmed using miRBase. Two separate pathway analyses were done for each cohort explored with NGS: OA vs C, OCD vs C and OA vs OCD blood plasma. Predictions were based off Tarbase v7.0 and were all experimentally validated targets of the DE miRNAs. When analysing the pathways of DE miRNAs in OA vs C blood plasma (Fig 1C), 22 KEGG pathways were identified to have potential interactions. Notable pathways included TGF-beta signaling pathway with 23 targeted genes by 3 miRNAs (FDR p = 0.0004), FoxO signaling pathway with 44 targeted genes by 3 miRNAs (FDR p = 0.0025), and Hippo signaling pathway with 39 targeted genes by 3 miRNAs. Next the potential affected pathways of DE miRNAs in OCD vs C plasma were investigated using the same methods (Fig 3C). 17 KEGG pathways were identified to have potential interactions. Hippo signaling pathway once again was identified with 56 potential targets by 5 miRNAs (FDR p = 1.76E^-9^). Additionally, ECM-receptor interaction was identified with 27 potential targets by 6 miRNAs (FDR p = < E^-325^), Gap junctions were identified with 10 targets by 2 miRNAs (FDR p = 0.0008), and adherens junctions were identified with 23 targets by 3 miRNAs (FDR p = 0.016). Affected pathways of DE miRNAs in OA vs OCD plasma were also assessed using the same technique described above (Fig 5C). 13 KEGG pathways were identified to have potential interactions. ECM-receptor interaction was identified as having 20 potential targets by 5 miRNAs (FDR p = 1.11E^-16^), Hippo signaling pathway with 38 potential targets by 2 miRNAs (FDR p = 0.0046), p53 signaling pathways with 26 potential targets by 2 miRNAs (FDR p = 6.038E^-5^), and proteoglycans in cancer with 65 potential targets by 3 miRNAs (FDR p = 1.63E^-12^).

## Discussion

OA and OCD are still detected only at the onset of clinical signs, but it is now know that multiple changes occur beforehand. There has been a growing effort to identify biomarkers that may be used pre-clinically to allow for earlier therapeutic intervention before clinical signs are present. Biofluids and specifically miRNAs have been poised as attractive candidates to use as biomarkers in either blood plasma or synovial fluid. MiRNAs have the additional benefit of being potential therapeutic targets by means of either anti-sense or mimetic therapies. The current study is among the first to implement NGS on the blood and synovial fluid from horses with and without OA or OCD to identify differentially expressed miRNAs. miRNAs and other small non-coding RNAs have previously been investigated in equine osteoarthritis [14,15]. When examining synovial fluid, miR-10a, miR-223, let-7a, miR-99a, miR-23b, miR-228, and miR-143 were found to be differentially expressed in early OA vs control [15]. In the current study, it was also found let-7a to be differentially expressed in OA vs C plasma and miR-223 to be differentially expressed in both OA and OCD vs C plasma. Taken together, this data suggests that plasma miR-223 and let-7a expression may be positive predictors of joint disease and warrants further investigation in future studies.

miR-196b was identified as significantly upgregulated in OA plasma through NGS. To much surprise, this finding was unable to be further validated with ddPCR in the plasma and synovial fluid samples. This may possibly be due to differences in sensitivity and chemistry between the NGS and ddPCR methods employed. Previous studies compared the accuracy and sensitivity with seemingly mixed results. NGS and ddPCR were shown to be highly agreeable in a study examining plasma EGFR mutations [18]. Additionally, a comparison of both techniques when examining human papillomavirus (HPV) detection found each to have similar sensitivities when examining plasma but when examined in oral rinse, NGS had a far superior sensitivity of 75% compared to 8.3% in ddPCR [19]. Lastly, NGS was shown to have higher sensitivity compared to ddPCR detecting *KRAS* mutations in liquid biopsy samples of patients with colorectal cancer [20]. Another explanation for the discrepancy between the NGS and ddPCR data could be due to miRNA isoforms which are further discussed below. Previously, miR-196b has been identified as upregulated in human rheumatoid arthritis [21].

MiR-181a was identified to be significantly upgregulated in the plasma of OA and OCD horses compared to C. The NGS was then validated data using ddPCR and observed that plasma miR-181a expression was decreased in OA compared to C and OCD groups. No significant difference in synovial fluid samples were observed possible due to high variance in the OA samples. Possible explanations for the discrepancy in techniques was previously discussed. MiR-181a has been linked to OA previously [13,22]. Nakamura *et al* previously identified miR-181a-5p as a mediator of cartilage degradation in human facet joint OA. Furthermore, they later showed that miR-181a-5p antisense oligionucleotide (ASO) were able to attenuate knee and facet joint OA in human and mouse rat models [13]. miR-181a-5p ASO was shown to attenuate the expression of cells positive for matrix metallopeptidase 12 (MMP12), poly ADP ribose polymerase p85 (PARP p85), collagen type 10 (COL10), and cleaved caspase. Furthermore, miR-181a has been shown to reduce oxidation resistance through selenocysteine insertion sequence-binding protein 2 (SECISBP2) [23]. and modulate chondrocyte apoptosis via glycerol-3-phosphate dehydrogenase 1 like (GPD1L) in OA [24].

MiR-140-5p has been extensively studied in regards to OA. The current study has shown that an increase in miR-140-5p expression in plasma of OCD horses vs C horses via NGS. Validation through ddPCR showed a significant decrease of expression in OA compared to both OCD and C plasma and demonstrated significant upgregulation in OA compared to both C and OCD synovial fluid. Previous studies have shown miR-140-5p to have a protective effect and attenuate OA. IA injections of miR-140 mimic in a destabilization of the medial meniscus (DMM) rat model showed a decrease of matrix metalloproteinase 13 (*MMP-13*) and *ADAMTS-5* (a disintegrin and metalloproteinase with thrombospondin motifs 5) expression levels [11]. In a previous study we have shown miR-140 to be involved in the chondrogenic differentiation of equine cord-blood mesenchymal stromal cells through the targeting of *ADAMTS-5*. Here it has been demonstrated, to the best of our knowledge, the first link of elevated miR-140 in OA.

The current study demonstrated miR-20a to be significantly increased in OA vs C plasma with NGS while ddPCR showed a decrease of expression in OA vs C and OCD. In synovial fluid miR-20a was demonstrated to be significantly increased in OA vs OCD samples. Possible explanations for the discrepancy in techniques were previously discussed. Previous studies have shown miR-20a to be elevated in OA cartilage tissues and synovial tissues [25–28]. miR-20a inhibition led to a reduction of NF-kB (nuclear factor kappa-light-chain-enhancer of activated B cells) pathway activity and chondrocyte apoptosis [28]. It has also been shown to be a key regulator of chondrogenic differentiation by suppressing autophagy via autophage related 7 (Atg7) [26].

Following NGS and ddPCR validation, we began to investigate predicted pathways of DE miRNAs based on the NGS data. Several signaling pathways were identified including Hippo signaling pathway and forkhead box O (FoxO) signaling pathway. Interestingly Hippo signaling pathway and its role in OA progression has been gaining interest in recent years. OA cartilage has been observed to exhibit increased Yes-associated protein (YAP) expression and YAP inhibition has been shown to prevent cartilage degradation. YAP overexpression causes an increase of MMP13 and ADAMTS-5 expression following interleukin 1 beta (IL-1B) insult. A previous study has also shown Hippo signaling pathway to be a top candidate of targeted pathways in human OA serum [29]. YAP has also been shown to be linked to NF-kB signaling by interacting TAK1 (transforming growth factor-β (TGF-β)-activated kinase 1) thereby suppressing NF-kB signaling through reduced substrate availability [30]. FoxO signaling has been identified as a mediator of transforming growth factor beta (TGF-B) and TAK1 signaling [31]. Loss of FoxO1 caused OA phenotypes to develop in cartilage whereas FoxO1 gain had a protective effect against induced OA models [31]. Additionally, FoxO family transcription factors have been identified to modulate proteoglycan 4 in articular cartilage and are essential in regulating cartilage homeostasis. In FoxO-deficient mice, proteoglycan 4 reduced expression was an initiating factor to structural changes in articular cartilage and contributed to the rapid destruction observed in the OA phenotype [32].

Interestingly, much of the NGS data was unable to be validated using ddPCR (Table 5). While one possible explanation for this observation was previously highlighted, isomiRs may also play a pivotal role in this discrepancy. IsomiRs are miRNA isoforms that have 5’ or 3’ end lengths that differ from those in miRNA data bases [33–36]. These slight changes in end lengths can be detected via NGS but detection via PCR techniques remains challenging as they require primers specific for each isomiR [33,36]. In NGS, the total read count of each miRNA often includes the registered canonical sequences as well as any 3’ and 5’ isomiRs [35,36]. Couple this with the growing body of evidence that isomiRs may be the most abundant form of miRNA expression and a likely explanation emerges of why ddPCR was unable to validate the NGS data in the current study. Recently, a study examined miR-1246 isomiRs in the serum of lung cancer patients. It found that in both healthy control and non-small cell lung cancer patients, miR-1246|-2|0| (an isomiR 2 base pairs shorter on the 5’ end) was more abundant [36]. Additionally, it was observed that miR-1246|-2|0| isomiR levels strongly correlated positively with total miR-1246 levels but this correlation was not observed when comparing total miR-1246 levels with that of the canonical sequence [36]. Another study found that over half of the miRNAs evaluated were expressed as isomiRs and not as the registered canonical sequence [35]. Although when selecting the miRNA primers used in ddPCR analysis it was ensured that the human and equine sequences matched, it seems likely that isomiRs were making up a major portion of the NGS reads. Future studies utilizing NGS should therefore examine isomiRs as well as the canonical sequence when validating sequencing data. A previous study examining miRNA expression in synovial fluid was able to partially validate NGS data with qRT-PCR data [15]. However, LNA primers have been shown to have improved specificity compared to conventional primers [37]. The combination of the current utilizing LNA primers combined with the improved sensitivity of ddPCR vs qRT-PCR may explain the discrepancy between studies in validating NGS results. Utilizing conventional primers with the less sensitive qRT-PCR protocol may downplay the issues that isomiRs play in validating NGS data. Future work should be done examining which technique is better suited to overcome the difficulties that isomiRs introduce in NGS validation.

**Table 5:** Summary of miRNAs selected for validation and their expression profiles compared in NGS vs ddPCR in equine blood plasma.

miRNA	NGS	ddPCR
eca-miR-20a	↑ OA vs C plasma	↓ OA vs C/OCD plasma
eca-miR-181a	↑ OA/OCD vs C plasma	↓ OA vs C/OCD plasma
eca-miR-196b	↑ OA vs C/OCD plasma	No Significance
eca-miR-486	↑ OA/OCD vs C plasma	↓ OA vs C/OCD plasma
eca-miR-140	↑ OCD vs C plasma	↓ OA vs C/OCD

It is recognized that a limitation of the current study are the samples used. Equine samples can be hard to come by, especially controls. The control cohort of horses were sourced from the Arkell Research Herd and as such were much older on average than both diseased cohorts as this cohort is mostly comprised of athletes. While we did our due diligence to omit any controls that had a history of joint disease as well as any obvious joint issues via a routine lameness examination, it is entirely feasible that these animals may have had underlying joint disease that could have skewed the results. Due to cost limitations, it was not feasible to further examine these horses with other techniques such as radiographs. Future follow-up studies are underway with younger control horses where a thorough lameness assessment including radiographs and synovial fluid analysis are being performed.

Age, sex, breed and hemolysis are possible influencers of miRNA expression. miRNAs have been shown to decrease in abundance with age within human plasma including miR-181a-5p [38]. The influence of sex on miRNA expression has also been previously investigated in a variety of diseases showing that expression changes exist between males and females [39]. These sex-dependent expression changes can be linked to either hormone regulation or sex chromosomes. Breed has also been shown to play a role in miRNA expression. A study examining expression in the skeletal muscle cells of cattle breeds revealed 23 miRNAs to be differentially expressed which influenced myotube formation [40]. Similarly, an equine study found 50 serum miRNAs as potential biomarkers of breed types [41]. Unfortunately, controlling for these variables was beyond the scope of the current study due to a very limited sample pool. Future studies may control for these variables as we continue to expand our biobank. From a clinical perspective, this studies approach to these variables may also prove useful in identifying so-called ‘smoking gun’ miRNAs that are differentially expressed irrespective of age, sex, and breed. These should be further validated in future studies controlling for these variables. Hemolysis may also impact miRNA expression within plasma [42]. miRNAs may be released into circulation by hemolyzed red blood cells thereby skewing results [42]. Of note, one study examined hemolyzed equine serum and found miR-486 to be highly influenced in these samples [43]. Additionally, when hemolyzed samples were excluded, the study found a marked reduction in differentially expressed miRNAs with 9 out of the original 121 miRNAs remaining [43]. In the current study, we did not take in to account the potential for hemolysis to impact miRNA expression. Future studies examining equine plasma should make this consideration to avoid the potential of skewed results.

### Conclusion

The current study has demonstrated a cohort of differentially expressed miRNAs in equine plasma for both OA and OCD using NGS. Additionally, DE miRNAs were able to be identified using ddPCR in synovial fluid (miR-140, miR-486, and miR-20a) based on the NGS findings. Several of the identified miRNAs are shared with human disease suggesting that there may be substantial overlap in the pathogenesis of the equine disease. Further investigation is warranted for the therapeutic use of these miRNAs for the treatment and prevention of OA. This study marks a starting point for future studies to further validate the potential use of miRNAs as biomarkers for joint health. Before we arrive on biomarkers, it must be emphasized that much larger study cohorts are required for validation. Through the continued growth of an equine biobank, we hope to expand on our study cohorts in future studies.

## Supporting information

S1 File RNA Seq count data. (XLSX)

S2 File DESeq2 analysis. (XLSX)

S3 File ddPCR count data. (XLSX)
